# Possible modification of Alzheimer’s disease by statins in midlife: interactions with genetic and non-genetic risk factors

**DOI:** 10.3389/fnagi.2014.00071

**Published:** 2014-04-23

**Authors:** Mitsuru Shinohara, Naoyuki Sato, Munehisa Shimamura, Hitomi Kurinami, Toshimitsu Hamasaki, Amarnath Chatterjee, Hiromi Rakugi, Ryuichi Morishita

**Affiliations:** ^1^Department of Clinical Gene Therapy, Graduate School of Medicine, Osaka UniversitySuita, Japan; ^2^Department of Geriatric Medicine, Graduate School of Medicine, Osaka UniversitySuita, Japan; ^3^Division of Vascular Medicine and Epigenetics, Department of Child Development, United Graduate School of Child Development, Osaka University Office for University-Industry CollaborationSuita, Japan; ^4^Department of Biomedical Statistics, Graduate School of Medicine, Osaka UniversitySuita, Japan

**Keywords:** statin, Alzheimer’s disease, prevention, Abeta, isoprenoids

## Abstract

The benefits of statins, commonly prescribed for hypercholesterolemia, in treating Alzheimer’s disease (AD) have not yet been fully established. A recent randomized clinical trial did not show any therapeutic effects of two statins on cognitive function in AD. Interestingly, however, the results of the Rotterdam study, one of the largest prospective cohort studies, showed reduced risk of AD in statin users. Based on the current understanding of statin actions and AD pathogenesis, it is still worth exploring whether statins can prevent AD when administered decades before the onset of AD or from midlife. This review discusses the possible beneficial effects of statins, drawn from previous clinical observations, pathogenic mechanisms, which include β-amyloid (Aβ) and tau metabolism, genetic and non-genetic risk factors (apolipoprotein E, cholesterol, sex, hypertension, and diabetes), and other clinical features (vascular dysfunction and oxidative and inflammatory stress) of AD. These findings suggest that administration of statins in midlife might prevent AD in late life by modifying genetic and non-genetic risk factors for AD. It should be clarified whether statins inhibit Aβ accumulation, tau pathological features, and brain atrophy in humans. To answer this question, a randomized controlled study using amyloid positron emission tomography (PET), tau-PET, and magnetic resonance imaging would be useful. This clinical evaluation could help us to overcome this devastating disease.

## INTRODUCTION

Alzheimer’s disease (AD) is the most common form of dementia. The dramatic increase of life expectancy has resulted in an increasing number of patients with AD, imposing social and economic burden (Alzheimer’s Association, 2013). The neuropathological hallmarks of the disease include accumulation of senile plaques, composed of β-amyloid (Aβ), and neurofibrillary tangles (NFTs), composed of hyper-phosphorylated tau protein. There is evidence supporting that Aβ initiates the pathogenesis of AD (Masters and Beyreuther, 2006; Ballard et al., 2011). However, the failure of clinical trials of “anti-Aβ” therapies indicates that the intervention should be applied earlier, because Aβ deposition commences decades before the development of clinical symptoms of AD (Burns, 2009; Jack et al., 2010).

Interestingly, a prospective cohort study suggested that use of hydroxymethylglutaryl coenzyme A (HMG-CoA) reductase inhibitors, or statins, which are widely prescribed for the treatment of hypercholesterolemic patients (Endo, 2008), decreases the incidence of AD by half. However, controversy exists regarding whether statins have therapeutic effects on AD. In this review, the potential effects of statins against AD are addressed by drawing upon previous basic and clinical studies. Statin use from midlife could prevent AD.

## DYSLIPIDEMIA, AD, AND STATINS

The first statin, named “compactin,” was discovered and isolated from microorganisms by a research team led by Dr. Akira Endo at Sankyo Co. in 1972 (Endo, 2008). His discovery initially suffered from the limitations of animal studies, because rodents were resistant to cholesterol reduction by statins (Endo, 2004). Dozens of studies using larger animals, like chicken, dog, and monkey, and a clinical study in familial hyperlipidemia patients showed that this statin reduces plasma low-density lipoprotein (LDL)-cholesterol level (Mabuchi et al., 1981). Today, several statins are now in clinical use. Statins reduce cholesterol level via the inhibition of HMG-CoA reductase in hepatic cells. Sterol regulatory element-binding proteins (SREBPs) sense this change in cholesterol level, with a subsequent increase in their LDL receptor (LDLR) expression to uptake serum LDL cholesterol in order to compensate for the reduced cellular cholesterol content (Goldstein and Brown, 2009). Then, clinical studies have shown that statins prevent cardiovascular and cerebrovascular events (Shepherd et al., 1995; Crouse et al., 1997; White et al., 2000; Heart Protection Study Collaborative Group, 2002) by reducing plasma cholesterol level (Amarenco and Labreuche, 2009; De Caterina et al., 2010) and regulating an isoprenoid pathway (Downs et al., 1998; Sever et al., 2003; Zhou and Liao, 2010). Of note, the preventative effects could be more evident in middle age (Shepherd et al., 1995, 2002; Heart Protection Study Collaborative Group, 2002).

### CLINICAL STUDIES OF STATINS AND AD

It is conceivable that statins prevent strokes, and thereby reduce the incidence of vascular dementia. However, further studies are required to clarify whether statins are effective to prevent AD. Firstly, two case–control studies supported the preventive effect of statins on AD (Jick et al., 2000; Wolozin et al., 2000), whereas the results of subsequent studies including prospective studies are controversial (Heart Protection Study Collaborative Group, 2002; Shepherd et al., 2002; Zamrini et al., 2004; Dufouil et al., 2005; Rea et al., 2005; Zandi et al., 2005; Sparks et al., 2008; Haag et al., 2009; Feldman et al., 2010; Sano et al., 2011; Bettermann et al., 2012). Two recent randomized clinical studies enrolling around 400 mild-to-moderate AD patients have failed to show benefits of atorvastatin and simvastatin on cognitive function (Feldman et al., 2010; Sano et al., 2011). Although these two randomized studies could refute the beneficial effects of statins on disease progression in AD patients, there were several limitations including the duration of intervention (18-month period) and the selection of subjects such as the exclusion of AD patients requiring treatment for dyslipidemia with lipid-lowering agents. On the other hand, several prospective studies indicate that statins could prevent the onset of AD (Jick et al., 2000; Sparks et al., 2008; Haag et al., 2009; Bettermann et al., 2012). Follow-up period and cohort size of such supportive prospective studies are relatively longer and larger: over 6 years of follow-up and more than 1000 subjects enrolled. A recent meta-analysis by Wong et al. (2013) also showed preventive effects of statins on AD. Of note, several reports indicated preventive effects of statins on AD only when they included relatively young subjects (Rockwood et al., 2002; Li et al., 2004, 2010a). Taken together, these results of previous clinical studies indicate that the preventive effects and therapeutic effects against AD should be separately considered to elucidate the effects of statins against AD. Such idea corresponds with current understanding of “anti-Aβ” therapies: once clinical symptoms develop, other mechanisms would be required to treat AD (Holmes et al., 2008; Burns, 2009). Therefore, possible benefits of statins against AD might be obtained if administered decades before the onset of the disease.

### DIFFERENCES AMONG STATINS

One important aspect to consider in the effects of statins is their pharmacodynamics and permeability into the brain (Butterfield et al., 2011). Brain-permeable statins, such as simvastatin and lovastatin, might readily act on neurons and glia in the brain, and exert neuroprotective effects including Aβ-lowering effects (Fassbender et al., 2001; Simons et al., 2002). However, controversy exists regarding whether brain-permeable statins could be beneficial for AD (Wolozin et al., 2000; Arvanitakis et al., 2008). Among the studies showing preventive effects of statins on AD, only sub-analysis in the Ginkgo Evaluation of Memory Study reported that lipophilic statins tended to reduce the risk of dementia more than did hydrophilic statins (Bettermann et al., 2012). Notably, in the Rotterdam study, which showed a risk reduction of AD by statins, there was no difference in this beneficial effect between hydrophilic and lipophilic statins (Haag et al., 2009). Although the results of these studies may suffer limitations of small numbers for distinguishing hydrophilic and lipophilic statin users, the results of the latter prospective study suggested that the possible preventive effects of statins on AD might be due to the common mechanism of action of statins rather than the specific mechanism of action of brain-permeable statins. A recent review by Shepardson et al. (2011) discussed the difficulty in attributing the protective effects of statins to specific brain-permeable statins, by pointing out the existence of several confounding effects, including the prescribed dosage, pattern and duration, statistical power, and genetic risks.

## NON-GENETIC RISK FACTORS, AD, AND STATINS

Non-genetic risk factors for AD include age, gender, physical activity, obesity, midlife hypertension, and diabetes mellitus. Meta-analyses have shown that the odds ratio of women to develop AD relative to men is 1.56 (95% CI 1.16–2.10; Jorm and Jolley, 1998). Thus far, no clinical report has recognized the gender-specific beneficial effect of statins on AD. A very high dose of lovastatin of 100 mg/kg/day, equal to around 100 times the human dose, increased Aβ deposits only in female Tg2576 mice (Park et al., 2003). Compared to men, women receiving statins may lack risk reductions in mortality and stroke (Dale et al., 2007). These preclinical and clinical studies imply that statins may also exert a gender-specific effect on AD.

### OBESITY, AD, AND STATINS

A recent meta-analysis showed that obesity increases the risk of AD [hazard ratio (HR) 1.80, 95% CI 1.00–3.29; Beydoun et al., 2008]. Also, it was observed that midlife obesity is associated with an increased risk of some types of cognitive deficit (Debette et al., 2011). Statins might have benefits on obesity by increasing the catabolism of several lipids as well as apolipoprotein B-100, a key player in central obesity (Watts et al., 2003; Ooi et al., 2008). Regulation of peroxisome proliferator-activated receptor-α through an isoprenoid pathway also seems to be important for such benefits of statins (Roglans et al., 2002; Landrier et al., 2004; Paumelle et al., 2006).

### HYPERTENSION, AD, AND STATINS

Several papers report that midlife hypertension is a risk for AD (Skoog et al., 1996; Launer et al., 2000). This is also supported by recent clinical studies showing that blood pressure-lowering therapy can reduce the risk of AD or dementia (Launer et al., 2010; Li et al., 2010b). A meta-analysis of randomized controlled trials showed that statins have beneficial effects on hypertension (Strazzullo et al., 2007). These effects are additive with those of anti-hypertensive drugs, and occur independently of the baseline and change in cholesterol levels. This suggests the involvement of pleiotropic effects against hypertension-induced systemic inflammation and oxidative insults through the regulation of an isoprenoid pathway, but not blood pressure lowering itself (Strazzullo et al., 2007). Further analysis of this effect might reveal a role of statins in the crosstalk between midlife hypertension and AD.

### DIABETES, AD, AND STATINS

A recent meta-analysis of 15 prospective cohort studies revealed that diabetes also increases the risk of AD (HR 1.39, 95% CI 1.16–1.66; Lu et al., 2009). Interestingly, midlife diabetes seems to accelerate brain aging such as hippocampal atrophy and cognitive decline (Nooyens et al., 2010; Debette et al., 2011). It is well known that statin treatment can reduce the risk of coronary artery disease events in patients with diabetes (Kearney et al., 2008). As the underlying mechanism, regulation of the receptor for advanced glycation end products, RAGE, an important mediator of oxidative and inflammatory processes, might be involved in such statin actions (Lanati et al., 2010; Takeda et al., 2011). Of note, RAGE could also be an important target for AD therapy (Deane, 2012). While a meta-analysis of sub-analysis showed that statins very weakly increase the risk of diabetes itself (HR 1.09, 95% CI 1.02–1.17; Sattar et al., 2010), J-PREDICT (Japan Prevention Trial of Diabetes by Pitavastatin in Patients with Impaired Glucose Tolerance), an only cohort study whose primary outcome was onset of diabetes, showed a reduction of incidence of diabetes in statin-treated group (Odawara et al., 2013). Therefore, these findings suggest that diabetes may lie as a confounding factor between statins and AD. Further analysis is required to access the effect of stain on the incidence of diabetes, then on that of AD in pre-diabetic and diabetic patients.

## MECHANISMS OF ACTION

### EFFECTS OF STATINS ON CHOLESTEROL

Several groups have reported that cholesterol-lowering drugs, besides statins, decrease Aβ production, while cholesterol itself increases Aβ production in cell culture and animal studies (Racchi et al., 1997; Simons et al., 1998; Refolo et al., 2000; Kojro et al., 2001; Shie et al., 2002; Wahrle et al., 2002). Therefore, cholesterol-lowering could have a possible preventive effect against the onset of AD, though the role of cholesterol in AD remains unknown in humans. First, it is controversial whether the systemic cholesterol level is relevant to the incidence or the progression of AD (Jarvik et al., 1995; Yoshitake et al., 1995; Notkola et al., 1998; Romas et al., 1999; Evans et al., 2000; Kivipelto et al., 2002; Tan et al., 2003; Sabbagh et al., 2004). Brain cholesterol or derivative level is also inconsistent among studies using AD brains (Svennerholm and Gottfries, 1994; Sparks, 1997; Mulder et al., 1998; Papassotiropoulos et al., 2002; Schonknecht et al., 2002; Teunissen et al., 2003; Vega et al., 2003; Cutler et al., 2004; Dietschy and Turley, 2004; Kölsch et al., 2004, 2010; Leoni et al., 2006; Vance et al., 2006). One of the aims of these observations is the determination of optimal cholesterol levels for the prevention of AD. Some studies reported that a high systemic cholesterol level or high-density lipoprotein (HDL) cholesterol level is associated with longevity and is also protective against AD development in very elderly people, suggesting different roles of cholesterol at different ages (Weverling-Rijnsburger et al., 1997; Reitz et al., 2010). It is noteworthy that some statins can increase plasma HDL level (Jones et al., 1998). On the other hand, a high midlife serum cholesterol level has been reported to be a risk factor for the incidence of AD or AD-related pathological changes (Kivipelto et al., 2002; Pappolla et al., 2003). This was confirmed by a recent meta-analysis (Anstey et al., 2008). Therefore, the possible preventive effects of statins on AD might be related to regulation of cholesterol metabolism in midlife.

One inevitable point that must be considered is that cholesterol itself seems to be very important for brain function and maintenance (Dietschy and Turley, 2004; Vance et al., 2006). In fact, a marked reduction of cholesterol levels by high doses of statins seems to decrease neurotropic support and prove toxic for neurons (Fan et al., 2001; Suzuki et al., 2004). This might correspond with some case reports and prospective studies observing adverse effects of statins, particularly brain-permeable simvastatin and strong cholesterol-lowering atorvastatin, on cognitive function in especially elderly patients with dementia (Wagstaff et al., 2003; Evans and Golomb, 2009; Padala et al., 2012). Following these reports, the U.S. Food and Drug Administration (FDA) recently added safety warnings to statins concerning confusion and memory loss. Such potential adverse effects on cognitive function should be clarified for the appropriate application of statins for AD prevention or treatment.

ApoE, one of the essential players regulating brain cholesterol metabolism, is the strongest genetic risk factor for AD (Ashford, 2004). The *APOE*-ε4 allele increases the accumulation of senile plaques in AD patients as well as in cognitively normal people (Reiman et al., 2009; Morris et al., 2010). In the periphery, apoE, mainly secreted from the liver, regulates systemic cholesterol metabolism, and is related to dyslipidemia and arteriosclerosis (Kolovou and Anagnostopoulou, 2007; Kolovou et al., 2008). In *APOE*-ε4 carriers, plasma LDL cholesterol levels are around 10% higher, due to differences in apoE distribution in cholesterol-lipid particles and the metabolism of apoE-containing lipid particles (Cullen et al., 1998; Lucic et al., 2007). Therefore, such increase of plasma cholesterol level in *APOE*-ε4 carriers might be a target for prevention of AD. In the brain, apoE plays important roles in brain function through lipid transport as HDL-like particles (Raber et al., 2000; Ji et al., 2003; Bu, 2009; Filippini et al., 2009; Verghese et al., 2011; Wisdom et al., 2011; Trachtenberg et al., 2012). We showed that statin treatment increased LDLR-related protein 1 (LRP1, one of the apoE receptors)-mediated clearance of Aβ (Shinohara et al., 2010). Interestingly, a prospective study reported that the risk reduction of AD by statins was observed only in subjects with the *APOE*-ε4 allele (Li et al., 2004). On the other hand, *APOE*-ε4 carriers seem to be less responsive to statins in the regulation on lipids (Hubacek and Vrablik, 2011). Therefore, these studies might suggest a possible action of statins on amyloid burden through modulation of the apoE-cholesterol pathway.

### PLEIOTROPIC EFFECTS OF STATINS

#### Oxidative stress, inflammatory stress, and statins

Oxidative stress and inflammation are thought to be tightly linked to the pathogenesis and development of AD (Zhu et al., 2007; Pratico, 2008; Bennett et al., 2009; Heneka et al., 2010; Imbimbo et al., 2010). More importantly, many epidemiological studies suggest that anti-oxidative drugs and anti-inflammatory drugs may reduce the risk of AD. One randomized clinical trial suggested that anti-oxidative drugs (vitamin E and selegiline) might slow the progression of AD (Sano et al., 1997). However, a recent randomized clinical trial failed to show any beneficial effect of vitamin E on the conversion of mild cognitive impairment (MCI) to AD (Petersen et al., 2005). Controversy also exists among the results of randomized prevention trials of anti-inflammatory drugs (Thal et al., 2005; Lyketsos et al., 2007; Gomez-Isla et al., 2008; Small et al., 2008). A randomized trial even reported an increased risk of AD using celecoxib and naproxen, a Cox-2 inhibitor, in people aged 70 years or older (Lyketsos et al., 2007). This implies that anti-inflammatory drugs may worsen AD by reducing some kind of beneficial inflammation for AD (Akiyama and McGeer, 2004; Wyss-Coray, 2006), or, as an elderly community-based cohort study suggested, the increased incidence of AD in later life in anti-inflammatory drug users during midlife may reflect delayed effects of the onset of AD (Breitner et al., 2009). Thus, it remains undetermined whether anti-oxidative or anti-inflammatory drugs are beneficial if administered years before the onset of AD, as with statins.

As noted earlier, several papers showed that an isoprenoid-dependent mechanism of statins is involved in their anti-oxidative and anti-inflammatory effects. Reactive oxygen species are attenuated via the regulation of NADPH by statins (Nakagami et al., 2003). Statins can also modulate several inflammatory responses via peroxisome proliferator-activated receptor-α and -γ and nuclear factor-kappa B (Liao and Laufs, 2004; Paumelle and Staels, 2007). In an AD animal study, simvastatin reduced oxidative stress and inflammation in amyloid precursor protein (APP) transgenic mice (Tong et al., 2009). It was also shown that fluvastatin reduced oxidative damage and ameliorated neuronal degeneration and cognitive dysfunction in AD model mice (Kurinami et al., 2008). These studies suggest that a possible mechanism of the preventive action of statins against AD involves anti-oxidative and anti-inflammatory effects via an isoprenoid-dependent pathway.

#### Effects of statins in vasculature

Vascular impairment is one aspect of AD. Pathologically impaired vessels have been observed in the brain of AD patients, as represented by cerebral amyloid angiopathy (CAA). The formation of CAA might be promoted by impairment of vascular flow (Thal et al., 2008; Weller et al., 2009). Furthermore, neurovascular coupling, which controls cerebral blood flow in response to neuronal function, is thought to be involved in the development of AD (Iadecola, 2004).

Dysfunction of neurovascular coupling can be reversed by anti-oxidative drugs and anti-inflammatory drugs (Iadecola, 2004). In the Rotterdam study, the HR of AD was reduced by all statins, regardless of their lipophilicity (Haag et al., 2009). Therefore, the preventive action of statins on AD could be due to their effects on brain blood vessels, as hydrophilic statins act more dominantly on vessels rather than cells inside the brain. Even a lipophilic statin, simvastatin, improved neurovascular dysfunction in an AD mouse model, by attenuating oxidative stress and inflammation. Importantly, such reversal of neurovascular dysfunction was associated with improvement of short- and long-term memory (Tong et al., 2009, 2012). An isoprenoid-dependent effect is involved in increasing the endothelial nitric oxide synthase (eNOS) level via the PI3K/Akt pathway, which is important for vascular function and maintenance (Zhou and Liao, 2010). Li et al. (2006) showed that simvastatin increased the levels of Akt, phosphorylated, Akt and eNOS, and reversed learning deficits in Tg2576 mice, independently of Aβ deposition. Kurata et al. (2012) also reported that atorvastatin and pitavastatin protected against degenerations of neurovascular units and reversed learning deficits in Tg2576 mice. In addition, regulation of systemic cholesterol by statins might play important roles on prevention of AD by the reduction of cerebrovascular atherosclerosis, which is reported to correlate with AD pathology, including senile plaques and CAA (Beach et al., 2007; Yarchoan et al., 2012). These studies suggest that one of the important actions of statins with regards to AD could involve blood vessels in the brain.

#### Aβ metabolism and statins

The Religious Orders Study, a longitudinal clinical pathologic study, observed that statin users, especially those using lipophilic statins, are less likely to have amyloid load in the brain of old Catholic clergy at autopsy (Arvanitakis et al., 2008). Another study also showed that cognitively normal people who used statins have fewer amyloid plaques at autopsy (Li et al., 2007). The mechanism by which statins regulate Aβ metabolism in the brain is still not fully clarified yet. As with other proteins, the level of Aβ is determined by the balance of its production and clearance. Blood vessels in the brain play an important role in the regulation of Aβ clearance (Bell and Zlokovic, 2009; Weller et al., 2009). Especially, LRP1 could be involved in Aβ clearance by brain blood vessels (Bell and Zlokovic, 2009). Our recent observations showed that fluvastatin increased Aβ clearance from the brain through up-regulating LRP1 level in the brain vessels of wild-type and APP transgenic mice. The notion that statins increase LRP1-mediated Aβ clearance was also supported by an *in vitro* model of Aβ clearance, using human brain blood vessel cells. In addition, an isoprenoid-dependent pathway mediates this effect (Shinohara et al., 2010). These results provide evidence that brain vessels may be directly involved in statins’ action on Aβ metabolism. Additional studies using conditional LRP1-knockout mice could clarify the involvement of LRP1 in the enhancement of Aβ clearance by statins (Kanekiyo et al., 2012). It was also shown that statins increased Aβ clearance by up-regulating insulin degrading enzyme, an Aβ-degrading enzyme *in vitro* (Tamboli et al., 2010). Taking these findings together, statins could restore Aβ clearance, which is reported to be decreased in AD patients (Mawuenyega et al., 2010).

Many groups also reported that statins regulate Aβ production, although different mechanisms are proposed. Possible mechanisms include regulation of shedding by α/β/γ-secretase, APP-trafficking, or degradation of APP-CTFs (C-terminal fragments of APP) through a cholesterol-dependent or isoprenoid-dependent mechanism (Kojro et al., 2001; Burns et al., 2006; Parsons et al., 2006; Ostrowski et al., 2007; Roensch et al., 2007; Won et al., 2008; Zhou et al., 2008; Guardia-Laguarta et al., 2009). Notably, these proposed mechanisms are all based on *in vitro* culture experiments or animal experiments with high doses of statins. Therefore, the effect of statins *in vivo*, at clinically relevant doses, has yet to be uncovered. Fluvastatin treatment at clinically relevant doses reduced Aβ production by up-regulating the lysosomal degradation of APP-CTFs in the brain of young C57BL/6 mice. This effect was mediated by an isoprenoid-dependent pathway, similarly to the up-regulation of LRP1. Changes in the intracellular distribution of APP-CTFs and small GTPases, Rab5 and Rab7 might be explored to further elucidate the mechanisms of this effect (Shinohara et al., 2010). This result seems to be important, as fluvastatin, one of the brain-impermeable hydrophilic statins, also can modulate the Aβ production pathway in the brain at a clinical dosage. This effect might be mediated by the partial inhibition of small GTPases, as Ostrowski et al. (2007) indicated.

#### Tau metabolism and statins

Another key target for AD might be NFTs, where tau is hyperphosphorylated and forms a filamentous structure. The degree of neuronal loss and the severity of dementia correlate with the accumulation of NFT rather than senile plaques in AD (Braak and Braak, 1991; Arriagada et al., 1992). Several types of dementia, known as tauopathies, also manifest NFT and neuronal loss (Dickson, 2009). Regardless of whether NFT is a subsequent, parallel or separate event to Aβ accumulation, NFT could be an important target for prevention and treatment of AD (Small and Duff, 2008; Jack et al., 2013). Statins were reported to reduce NFT more significantly than senile plaques in cognitively normal subjects (Li et al., 2007). This observation suggests that statins could prevent tau accumulation more readily than Aβ accumulation.

Impaired cholesterol metabolism as well as oxidative and inflammatory stress seems to be involved in tau hyperphosphorylation (Schneider et al., 2004; Kitazawa et al., 2005; Michikawa, 2006; Ohm and Meske, 2006; Maccioni et al., 2010). Atorvastatin suppressed tau hyperphosphorylation induced by excess cholesterol in the rat brain (Lu et al., 2010). On the other hand, Boimel et al. (2009) found that simvastatin and atorvastatin reduced NFT and improved cognitive impairment in a tauopathy mouse model, associated with reduced inflammation. However, of note, cell culture studies showed that high doses of statins cause tau phosphorylation and cell toxicity (Hoglund et al., 2005b; Anstey et al., 2008). These findings suggest that optimal doses of statins may prevent tau hyperphosphorylation and NFT accumulation via both cholesterol- and isoprenoid-dependent effects. Although a study reported that 40 mg/day simvastatin decreased the phospho-tau level in patients with dyslipidemia without dementia (Riekse et al., 2006), other studies reported that statins including 20–80 mg/day simvastatin failed to show a decrease of phospho-tau or total tau level in the cerebrospinal fluid (CSF) in AD patients (Sjogren et al., 2003; Hoglund et al., 2005b; Serrano-Pozo et al., 2010) and middle-aged adults with risk of AD (Carlsson et al., 2008). Taken together, further clinical studies should determine the optimal doses of statins, taking individual disease stage into account.

#### Other genetic factors and statins

In addition to the *APOE* gene, recent genome-wide association studies (GWAS) identified novel risk genes for AD (Olgiati et al., 2011). These genes include box-dependent myc-interacting protein 1 (BIN1), clusterin (CLU, also called apolipoprotein J), ATP-binding cassette transporter A7 (ABCA7), complement component receptor 1 (CR1), phosphatidylinositol-binding clathrin assembly protein (PICALM), and CD33. As BIN1 and PICALM are involved in intracellular trafficking at synapses, these genes might modulate APP trafficking or tau metabolism (Xiao et al., 2012; Chapuis et al., 2013). CR1 and CD33 are key molecules in inflammatory cells, such as microglia (Crehan et al., 2012; Griciuc et al., 2013). CLU and ABCA7 are thought to play important roles in lipid homeostasis (Tanaka et al., 2011b; Yu and Tan, 2012). Kim et al. (2013) showed that ABCA7 inactivation in macrophages reduced phagocytic clearance of Aβ and exaggerated Aβ accumulation in mice. Interestingly, statins are reported to enhance ABCA7-dependent phagocytosis through the SREBP pathway (Tanaka et al., 2011a). These studies suggest crosstalk between statins and ABCA7 in Aβ metabolism. As these genetic factors are involved in Aβ metabolism, tau metabolism, inflammation, and lipid metabolism, statins may also have crosstalk with these molecules.

## FURTHER STUDY OF PREVENTIVE EFFECT OF STATINS FOR AD

Whether or not statins are effective for preventing AD is yet to be confirmed. Our study showed a modest effect of a statin on Aβ metabolism in C57BL/6 mice and APP transgenic mice (around 20% reduction of Aβ level in brain; Shinohara et al., 2010). We also observed that 3 months’ treatment with fluvastatin (before abundant Aβ plaque deposition) ameliorated impairment of spatial learning performance in APP/PS1 mice (Shinohara et al., unpublished results). These effects are most likely mediated by an isoprenoid-dependent pathway (Li et al., 2006). As cholesterol level is not readily affected by statins in rodents (Endo, 2008), larger animal models would also be required to demystify the cholesterol-dependent effects of statins (Murphy et al., 2010).

A surrogate marker for the regulation of Aβ metabolism as well as other AD-associated abnormalities would help to successfully establish preventive effects on AD. Our preliminary animal studies failed to show that statin treatment affects Aβ levels in the blood and CSF (unpublished data), whereas brain Aβ level was reduced by a statin (Shinohara et al., 2010). These experimental results are consistent with the results of clinical studies, reporting no change in Aβ levels in CSF and plasma by statin use in AD patients and cognitively normal adults (Ishii et al., 2003; Sjogren et al., 2003; Hoglund et al., 2004, 2005a; Riekse et al., 2006; Carlsson et al., 2008; Serrano-Pozo et al., 2010). These results suggest that Aβ levels in CSF and plasma would not reflect the “anti-Aβ” effect of statins in the brain. Therefore, amyloid- and tau-PET (positron emission tomography) imaging (Maruyama et al., 2013; Weiner et al., 2013), and other potential CSF surrogate markers like soluble form of LRP1 (Sagare et al., 2007, 2011) – might be helpful. Several markers of oxidative stress, inflammation, or vascular plasticity and integrity could be assessed to define the beneficial preventive action of statins against AD.

As mentioned above, statins might transiently impair cognitive function (Orsi et al., 2001; King et al., 2003; Wagstaff et al., 2003), especially where the drug is firstly administered to treat patients aged over 75 years. This adverse effect remains a rare occurrence (Rojas-Fernandez and Cameron, 2012). Considering their beneficial effects, statins should be used with close attention to the emergence of adverse effects in elderly patients.

## CONCLUSION

**FIGURE 1:**
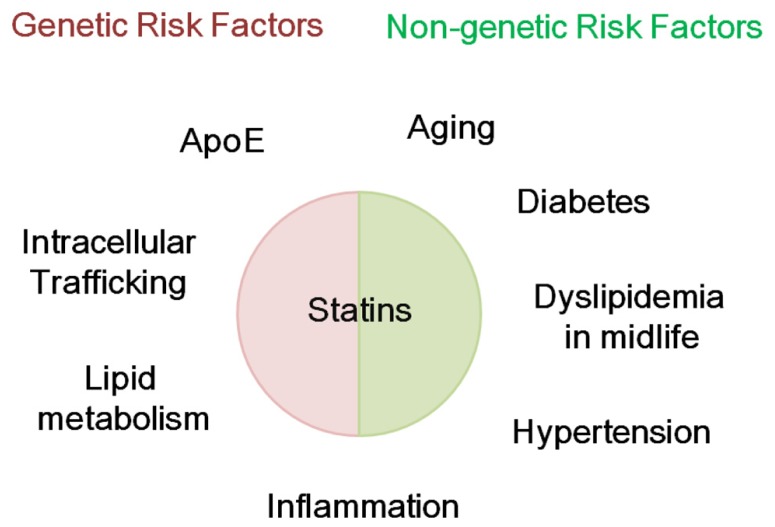
**Possible modification genetic and non-genetic risk factors for AD by statins.** Statins might modify genetic and non-genetic risk factors for Alzheimer’s disease (AD). Genetic risk factors for AD include apolipoprotein E and genes involved in intracellular trafficking, lipid metabolism, and inflammation. Non-genetic risk factors include aging, diabetes, dyslipidemia in midlife, hypertension, and inflammation.

This review describes the probable preventive actions of statins on AD. These effects could be achievable if statins were administered decades before the onset of AD or from midlife. Such effects, if present at all, could be negligible, with a 10–50% reduction in risk being expected from the positive results of clinical studies. The preventive effects of statins on AD include the regulation of Aβ metabolism, tau metabolism, cholesterol metabolism, oxidative and inflammatory stress, and vascular plasticity and integrity, all of which are independently/interdependently involved in the pathogenesis of AD (**Tables 1** and **2**). At least some of these effects could be mediated by regulation of the isoprenoid pathway. Therefore, exploring the mechanisms by which isoprenoid metabolism influences AD pathogenesis may clarify the isoprenoid-dependent effects of statins on AD (Eckert et al., 2009). Overall, these findings reviewed here suggest that administration of statins in midlife might prevent AD in late life by modifying genetic and non-genetic risk factors for AD (**Figure 1**). However, it still remains unclear whether statins really prevent AD in prospective well-controlled clinical studies. At least, it should be clarified whether statins inhibit Aβ accumulation, tau pathological features and atrophy in humans. To answer this question, a randomized controlled study using amyloid PET, tau-PET, and magnetic resonance imaging (MRI) would be useful. This clinical evaluation could help us to overcome this devastating disease.

**Table 1:** Summary of presumable beneficial actions of statin use in midlife on AD epidemiological factors.

		Targets of statins	
	Clinical evidence	Cholesterol pathway	Isoprenoid pathway
Hyperlipidemia	High cholesterol in midlife is a risk for AD (Kivipelto et al., 2002; Pappolla et al., 2003; Anstey et al., 2008)	Plasma LDL-cholesterol level and HDL cholesterol level (Jones et al., 1998; Goldstein and Brown, 2009)	–
*APOE*-ε4	Promotion of Aβ accumulation and down-regulation of brain activity (Raber et al., 2000; Ji et al., 2003; Bu, 2009; Filippini et al., 2009; Reiman et al., 2009; Morris et al., 2010; Verghese et al., 2011; Wisdom et al., 2011; Trachtenberg et al., 2012)	Plasma LDL-cholesterol (Raber et al., 2000)	–
Obesity	Obesity is a risk for AD, and midlife obesity promotes brain aging (Dale et al., 2007; Beydoun et al., 2008)	Plasma LDL, HDL, and apoB (Watts et al., 2003; Ooi et al., 2008)	PPAR-α (Roglans et al., 2002; Landrier et al., 2004)
Hypertension	Midlife hypertension is a risk for AD (Skoog et al., 1996; Launer et al., 2000)	–	eNOS, endothelin-1, and ROS (Strazzullo et al., 2007)
Diabetes	Diabetes is a risk for AD, and also midlife diabetes promotes brain aging (Lu et al., 2009; Nooyens et al., 2010; Debette et al., 2011)	–	RAGE, PPAR-α, -γ, NF-κB, and ROS (Lanati et al., 2010; Takeda et al., 2011)

*
Aβ, β-amyloid peptides; AD, Alzheimer’s disease; apoB, apolipoprotein B-100; apoE, apolipoprotein E; eNOS, endothelial nitric oxide synthase; HDL, high-density lipoprotein; LDL, low-density lipoprotein; NF-κB, nuclear factor-κB; PPAR, peroxisome proliferator-activated protein; RAGE, receptor for advanced glycation end products; ROS, reactive oxygen species.*

**Table 2:** Summary of presumable beneficial actions of statin use in midlife on AD pathogenic stress and molecules.

	Clinical evidence	Targets of statins	
		Cholesterol pathway	Isoprenoid pathway
Oxidative stress and inflammatory stress	Oxidative stress and inflammatory stress are involved in AD	–	Rac1-NADPH and PPAR-α, -γ and NF-κB (Nakagami et al., 2003; Liao and Laufs, 2004; Paumelle and Staels, 2007; Kurinami et al., 2008; Tong et al., 2009)
Vascular system	Midlife vascular risk factors potentiate development of AD	Cholesterol-induced cerebrovascular atherosclerosis	PI3K/Akt/eNOS pathway (Li et al., 2006; Zhou and Liao, 2010; Tong et al., 2012)
Tau accumulation	Tau starts to accumulate decades before the onset of AD (Jack et al., 2013)	Cholesterol-induced tau hyperphosphorylation (Lu et al., 2010)	Cholesterol-independent anti-inflammatory effects (Boimel et al., 2009)
Aβ accumulation	Aβ starts to accumulate decades before the onset of AD (Jack et al., 2010)	Cholesterol-involving Aβ production (Racchi et al., 1997; Simons et al., 1998; Refolo et al., 2000; Kojro et al., 2001; Shie et al., 2002; Wahrle et al., 2002)	APP-CTFs degradation via Rho or Rab family, and Aβ clearance via LRP1 or IDE (Ostrowski et al., 2007; Shinohara et al., 2010; Tamboli et al., 2010)

*Aβ, β-amyloid peptides; AD, Alzheimer’s disease; APP-CTFs, C-terminal fragments of amyloid precursor protein; eNOS, endothelial nitric oxide synthase; IDE, insulin degrading enzyme; LRP1, LDLR-related protein 1; NF-κB, nuclear factor-κB; PPAR, peroxisome proliferator-activated protein; Rac1, Ras-related C3 botulinum toxin substrate 1; NADPH, nicotinamide adenine dinucleotide phosphate; PI3K, phosphatidylinositol-4,5-bisphosphate 3-kinase; Akt, v-akt murine thymoma viral oncogene homolog.*

### SEARCH STRATEGY AND SELECTION CRITERIA

References for this Review were identified through searches of PubMed up to September 2013, with the search terms “((statins) OR statin) AND Alzheimer’s disease,” “((statins) OR statin) AND stroke,” “((statins) OR statin) AND Abeta,” “((statins) OR statin) AND tau,” “((statins) OR statin) AND cholesterol,” “((statins) OR statin) AND ((apolipoprotein E) OR apoE),” “((statins) OR statin) AND aging,” “((statins) OR statin) AND ((gender) OR sex),” “((statins) OR statin) AND obesity,” “((statins) OR statin) AND hypertension,” “((statins) OR statin) AND diabetes,” “((statins) OR statin) AND isoprenoid,” “((statins) OR statin) AND ((oxidative) OR inflammatory),” “((statins) OR statin) AND (((cognitive function) OR brain) OR memory),” “((statins) OR statin) AND ((vasculature) OR vascular),” and “((statins) OR statin) AND ((((((BIN1) OR PICALM) OR CD33) OR CR1) OR CLU) OR ABCA7).” Only papers published in English were reviewed. Clinical studies as well as animal or cell culture studies were included. Evidence independently reported by several studies (e.g., meta-analysis) was more emphasized than evidence reported by a single study.

## Conflict of Interest Statement

The authors declare that the research was conducted in the absence of any commercial or financial relationships that could be construed as a potential conflict of interest.
